# Ningxiang Pig-Derived Microbiota Affects the Growth Performance, Gut Microbiota, and Serum Metabolome of Nursery Pigs

**DOI:** 10.3390/ani14172450

**Published:** 2024-08-23

**Authors:** Hongkun Li, Li Han, Feng Zhou, Zichen Wu, Longlin Zhang, Renjie Xie, Feng Jiang, Qiyu Tian, Xingguo Huang

**Affiliations:** 1 College of Animal Science and Technology, Hunan Agricultural University, Changsha 410128, China; lhko328@163.com (H.L.); rosehan008@gmail.com (L.H.); i_zhoufeng@stu.hunau.edu.cn (F.Z.); zichen123@stu.hunau.edu.cn (Z.W.); zllwithann@gmail.com (L.Z.); xrj101900@foxmail.com (R.X.); jf17363914513@163.com (F.J.); 2 Yuelushan Laboratory, Changsha 410128, China; 3 Hunan Agriculture Research System, Changsha 410128, China

**Keywords:** pigs, fecal microbiota transplantation, growth performance, serum metabolism, gut microbiota

## Abstract

**Simple Summary:**

Gut microbiota has become a key factor affecting various phenotypic characteristics of the host. Chinese indigenous pig breeds (Ningxiang pigs) have shown greater stress resistance compared to commercial breeds (Duroc × Landrace × Yorkshire (DLY) pigs) which may be related to their distinctive gut microbiota. However, there are few studies on how exogenous microbiota (Ningxiang pigs) change the physiology or metabolism of the host. This study aimed to investigate the effects of fecal microbiota transplantation (FMT) from Ningxiang pigs on the growth performance, fecal microbiota, and serum metabolites of the same-old DLY pigs. The results showed that Ningxiang pig’s FMT could alleviate oxidative stress and enhance growth performance in DLY pigs by modulating their gut microbiota and metabolic features.

**Abstract:**

The gut microbiota is crucial for maintaining the host’s intestinal homeostasis and metabolism. This study investigated the effects of fecal microbiota transplantation (FMT) from Ningxiang pigs on the growth performance, fecal microbiota, and serum metabolites of the same-old DLY pigs. The results indicated that the average daily gain of FMT pigs was significantly greater than that of the control (CON) group. Compared to the CON group, the FMT group significantly improved the apparent digestibility of crude fiber, crude ash, gross energy, and calcium of the pigs. The analysis of serum antioxidant status revealed that the activities of total superoxide dismutase and catalase in the serum of pigs in the FMT group were significantly elevated, whereas the level of malondialdehyde was significantly reduced. Furthermore, 16S rRNA sequencing analysis revealed that the Ningxiang pig-derived microbiota altered the fecal microbiota structure and modulated the diversity of the gut microbiota in the DLY pigs. Untargeted LC–MS metabolomics demonstrated that pigs in the FMT group exhibited distinct metabolomic profiles compared to those in the CON group. Significant changes were observed in key metabolites involved in amino acid, lipid, and carbohydrate metabolism. Additionally, a correlation analysis between serum differential metabolites and the gut microbiota revealed that the relative abundance of *Lachnospiraceae_NK4A136_group* and *Corynebacterium* was highly correlated with lipid compounds. In conclusion, Ningxiang pig-derived microbiota can alleviate oxidative stress and enhance growth performance in DLY pigs by modulating their gut microbiota and metabolic features.

## 1. Introduction

Countless microorganisms inhabit the intestines of mammals. They play a crucial role in maintaining intestinal homeostasis, regulating the immune system, and influencing energy metabolism [1]. Consequently, the gut microbiota is also referred to as the “second genome” [2]. Research on humans and animals indicates that microbiota dysbiosis is associated with various acute and chronic inflammatory conditions, intestinal diseases, and metabolic syndromes [3]. In recent years, numerous studies have demonstrated that probiotics can directly or indirectly enhance animal health. However, due to the different sources and compositions of probiotics, they may not be able to adapt to the dynamic intestinal environment and therefore cannot colonize the intestines [4]. Fecal microbiota transplantation (FMT) involves transferring gut microbiota from healthy donors to recipients, with an aim to restore and preserve their gut microbiota [5]. Additionally, research indicates that FMT offers benefits in enhancing pig growth performance, elevating feed efficiency, and decreasing diarrhea rates, alleviating inflammatory bowel disease (IBD) [6,7,8,9]. Hence, FMT represents a potential approach for remodeling the gut microbiota, thereby improving animal health.

Indigenous pig breeds of China have developed unique gut microbial structures that are tailored to their environmental and dietary needs, representing a valuable resource. The Ningxiang pigs are known for their dietary fiber tolerance, strong adaptability, and high disease resistance [10,11]. As a domestic breed of livestock, the Ningxiang pig’s bacterial communities are more diverse than those of commercial breeds [12]. It has been reported that differences in gut microbiota between different pig breeds can affect host phenotypes, such as diarrhea rate or growth differences [6,13]. However, there are few studies on how exogenous microorganisms (Ningxiang pigs) change the physiology or metabolism of the host. Therefore, this experiment used DLY pigs as a model to explore the impact of exogenous fecal microbiota on animal health, focusing on growth performance, serum metabolism, and gut microbiota. Our findings could help us to understand the beneficial effects of microorganisms in Ningxiang pigs, to further select targeted microorganisms or microbiota-derived metabolites, ultimately enhancing animals’ stress resistance and improving their growth performance.

## 2. Materials and Methods

### 2.1. Experimental Design

Thirty-six healthy “Duroc × Landrace × Yorkshire” (DLY) pigs (purchased from Liu’an Agricultural Technology Comprehensive Development Co., Ltd., Changsha, China) were selected, with an average weight of 20 kg, an age of about 50 days, and a uniform genetic background. These pigs were randomly divided into two groups, the CON group and FMT group. In addition, 6 Ningxiang pigs (purchased from Liu’sha’he Ningxiang pigs Ecological Animal Husbandry Co., Ltd., Changsha, China) with similar weights to the DLY pigs were selected as the fecal bacteria donor group and raised in a separate column.

All the pigs in this experiment were fed the same basic diet (Table S1), which met the nutritional requirements recommended by the National Research Committee (NRC, 2012, https://nap.nationalacademies.org/catalog/13298/, accessed on 8 July 2024) for growing pigs. While the CON group was fed a basic diet, the FMT group added 10 mL of prepared Ningxiang pig’s fecal bacterial suspension to the basic diet once a day. The preparation method of Ningxiang pig’s fecal microbiota suspension was to collect fresh Ningxiang pig’s feces every morning, with about 10 g of feces collected from each pig. After mixing the feces, they were placed into six 50 mL centrifuge tubes, and then 40 mL of physiological saline was added to each tube. The feces and physiological saline water were thoroughly mixed using a vortex shaker, and then centrifuged at 3000 rpm for 5 min. The fecal suspension obtained after centrifugation was filtered using a sterile gauze to obtain the fecal bacterial suspension. The experiment lasted for 35 days, with a 7-day adaptation period. All the pigs were free to feed and drink. Other feeding management and immunization procedures were carried out in accordance with the animal protection and welfare regulations of the College of Animal Science and Technology, Hunan Agricultural University.

### 2.2. Sample Collection

First, 100 g of feed was obtained using the method of coning and quartering then dried in a constant temperature oven at 65 °C. After drying, the sample was crushed and then filtered through a 40-mesh sieve, packaged in Ziplock bags, and stored in a −20 °C refrigerator for nutrient content determination.

On day 32 of the experiment, in each group, ten pigs were selected for their weights, which were close to the average. Fecal samples were collected continuously for 3 days from the same 10 pigs in each group. From 9:00 a.m. to 6:00 p.m., the fecal samples were collected every hour. Then, 10% diluted sulfuric acid was added to the collected fecal samples and they were stored in a −20 °C refrigerator. The fecal samples collected from the same pig over three days were mixed, and an appropriate amount of each fecal sample was taken and dried in a constant temperature oven at 65 °C, flipping every 2 h during the drying process. After drying, the sample was crushed and then filtered through a 40-mesh sieve, packaged in Ziplock bags, numbered, and stored in a −20 °C refrigerator for nutrient content determination.

The same 10 pigs were fasted and the blood was collected from the anterior vena cava on the last day of the experiment in each group. The blood samples were placed in a vacuum anticoagulant tube and allowed to stand for 30 min. After centrifugation (4 °C, 3000 rpm for 15 min), serum was obtained. The serum was then separated and stored in a new cryopreservation tube at −80 °C for antioxidant index detection and untargeted metabolomics analysis. Fresh feces were collected from the same 10 pigs during defecation and placed in sterile cryopreservation tubes. They were quickly frozen in liquid nitrogen and stored at −80 °C for the detection of fecal microbiota.

### 2.3. Measurement of Apparent Total Tract Digestibility

According to Chinese national standards, the contents of acid insoluble ash (AIA), gross energy (GE), ether extract (EE), crude protein (CP), crude fiber (CF), ash (Ash), calcium (Ca), and total phosphorus (P) in feed and fecal samples were determined, along with the moisture content and an indirect calculation of the dry matter (DM) content. The apparent total tract digestibility (ATTD) of nutrients was calculated using the endogenous indicator AIA method. The calculation formula is as follows:

ATTD (%) = (100% − A1/A2 × F2/F1 × 100%)

A1 represents the AIA content in the feed, A2 represents the AIA content in the fecal matter; F1 represents the nutrient content in the feed, and F2 represents the nutrient content in the fecal matter.

### 2.4. Measurement of Antioxidant Indicators

The determination of total antioxidant capacity (T-AOC, A015-1-1), total superoxide dismutase (T-SOD, A001-1-1), malondialdehyde (MDA, A003-1-1), and Catalyst (CAT, A007-1-1) was carried out using commercial assay kits (NJJCBIO, Nanjing, China) and strictly followed the instructions provided.

### 2.5. DNA Extraction, 16S rRNA Sequencing of Fecal Microbiota and Data Analysis

A DNA extraction kit (Accurate Biology, Changsha, China) was used to extract the total genomic DNA of the fecal microbiota. The DNA quality was then detected with 1% agarose gel electrophoresis, and the purity and concentration of DNA was detected using the NanoDrop2000 (Thermo Scientific, Waltham, MA, USA). The extracted DNA was utilized as a template for PCR amplification of the V3–V4 variable region of the 16S rRNA gene. This process involved the use of the upstream primer 338F (5′-ACTCCTACGGAGGCAGCAG-3′) and the downstream primer 806R (5′-GACTACHVGGGTWTCTAAT-3′), which carried barcode sequences. The PCR reaction was conducted using TransGen AP221-02, TransStart Fastpfu DNA Polymerase, in a 20 μL reaction system. The PCR products of the same sample were mixed and 2% agarose gel was used for recovery. Then, the AxyPrep DNA Gel Extraction Kit (Axygen Biosciences, Union City, CA, USA) was used to purify the recovered products. Next, 2% agarose gel electrophoresis and the Quantus ™ Fluorometer (Promega, Madison, WI, USA) were used to detect and quantify the recovered products and build a library of purified PCR products.

Fastp (version 0.19.6) and FLASH (version 1.2.11) software were used for quality control and merging of the paired-end original sequencing data. This included filtering, denoising, merging, and the removal of non-chimeric sequences. The sequences were clustered into operational taxonomic units (OTUs) at a 97% similarity threshold using the UPARSE software (version 7.1). The RDP classifier (version 2.11) was used to align representative OTU sequences with the Silva 16S rRNA gene database (version 138) for taxonomic annotation, maintaining a confidence threshold of 70%. Majorbio (https://cloud.majorbio.com/, accessed on 13 March 2024) was utilized to calculate alpha diversity indices such as Ace, Chao 1, Shannon, and Simpson, principal coordinates analysis (PCoA), and linear discriminant analysis (LDA) effect size (LEfSe) to identify bacterial taxa with significant differences in genus-level abundance between groups (LDA score > 2, *p* < 0.05).

### 2.6. Untargeted Metabolomics Study of Serum

Add 400 μL of extraction solution prepared in equal proportions of acetonitrile and methanol to 100 μL of serum sample. After thoroughly mixing with a vortex instrument, ultrasonically extract the sample at a low temperature for 30 min. Subsequently, let the sample stand at −20 °C for 30 min, then centrifuge (4 °C, 13,000 rpm, 15 min). Discard the supernatant, blow dry with nitrogen, and add 120 µL of a resuspension prepared in equal proportions of acetonitrile and water. Perform ultrasonic extraction at 5 °C for 5 min, then centrifuge again (4 °C, 13,000 rpm, 5 min). Finally, transfer the supernatant to a new centrifuge tube for subsequent analysis. Mix all samples equally in volume to prepare quality control (QC) samples. During analysis, insert a QC sample every 5 serum samples to examine the repeatability of the entire analysis process. Measure the relative concentrations of serum metabolites using the UPLC TripleTOF system (AB SCIEX) in both positive and negative ion modes. After the detection is complete, import the obtained raw data into the metabolomics processing software Progenesis QI (version 3.0) (Waters Corporation, Milford, CT, USA) for data preprocessing to obtain the final data matrix for subsequent analysis. Next, use HMDB (https://hmdb.ca/, accessed on 19 February 2024) and Metlin (https://metlin.scripps.edu/, accessed on 19 February 2024) to annotate the database to obtain metabolite information. Upload the preprocessed data to Majorbio (https://cloud.majorbio.com/, accessed on 25 February 2024) to perform principal component analysis (PCA) and orthogonal partial least squares discriminant analysis (OPLS−DA). Screen the differential metabolites based on Variable Importance in Projection (VIP) > 1 and *p* < 0.05. Using the KEGG (https://www.genome.jp/kegg/, accessed on 26 February 2024) database, annotate and enrich the differential metabolites for metabolic pathway analysis.

### 2.7. Statistical Analysis

The data for this experiment were preliminarily organized using Excel 2021 (Microsoft, Washington, DC, USA) and analyzed using SPSS 27.0 (IBM, Chicago, IL, USA). An independent sample t-test was used for significance analysis, and a two-tailed test was used for the significance test. The results were expressed as means ± SEM, with *p* < 0.05 indicating significant difference and *p* < 0.01 indicating extremely significant difference.

## 3. Results

### 3.1. Growth Performance, Apparent Digestibility, and Serum Antioxidant Activity

The final body weight and average daily gain (ADG) of FMT group pigs were higher than those in the CON group after 28 days of FMT (Table 1). The ADG of the FMT group pigs was significantly higher than that of the CON group (*p* < 0.05), and the ADG increased by 21.76% compared with the CON group, while the final body weight of the FMT group pigs tended to increase compared with the CON group (*p* = 0.055). Compared to the CON group, FMT group increased the apparent digestibility of Ash, CF, Ca, and GE in the pigs’ feces (Table 2). In addition, we measured the serum oxidative stress biomarkers. The results show that, compared with the CON group, the FMT group significantly upregulated the activities of CAT (*p* < 0.05) and T-SOD (*p* < 0.05). At the same time, the levels of MDA (*p* < 0.05) were significantly reduced. However, there was no discernible variation in the activity of T-AOC (*p* > 0.05) (Table 3).

### 3.2. Ningxiang Pig-Derived Microbiota Shaped the Gut Microbiome in DLY Pigs

**Figure 1:**
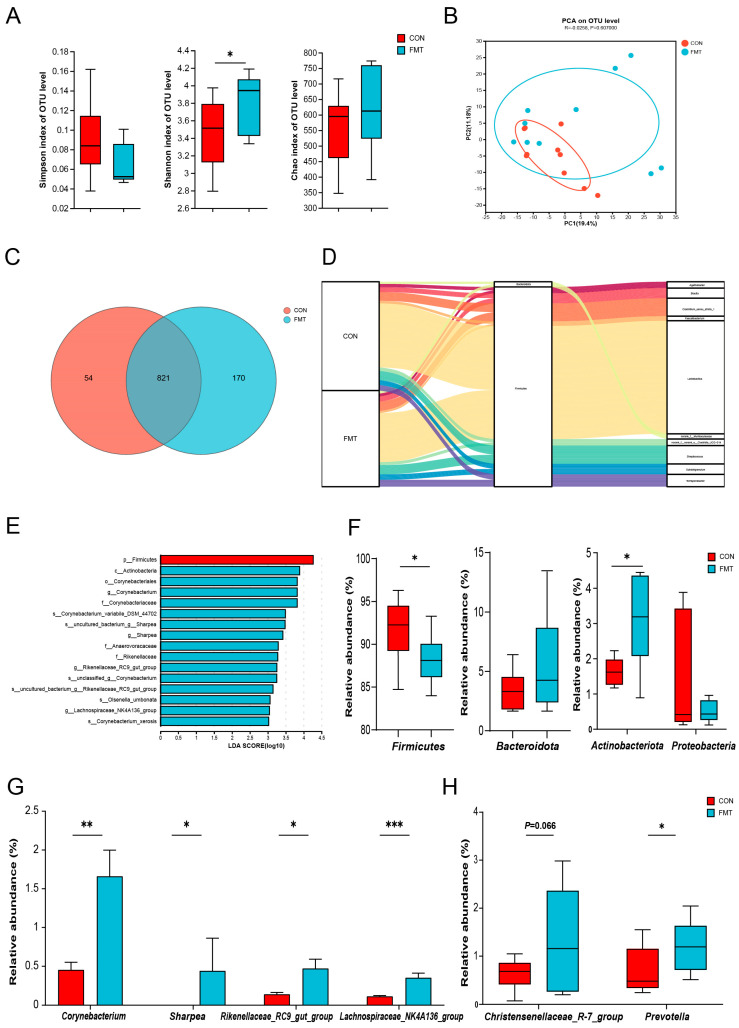
The effect of transplanting Ningxiang pig’s fecal microbiota on the diversity of fecal microbiota in DLY pigs. (**A**,**B**) DLY fecal microbiota Alpha diversity and Beta diversity (**A**) Alpha diversity: Simpson index, Shannon index, Chao 1 index. (**B**) Beta diversity: Principal Components Analysis (PCA) based on OTU levels. (**C**) Venn diagram: Common OTU analysis. (**D**) The Sankey plot displays the composition of the microbiota at the phylum and genus levels. (**E**) Screen community with LDA Score > 3 form kingdom level to genus level. (**F**−**H**) Comparison of differences in the relative abundance of microorganisms at the phylum and genus level. The statistical results are shown as means ± SEM (*n* = 10). (* *p* < 0.05 ** *p* < 0.01 *** *p* < 0.001).

We further analyzed the changes in microbial composition of the feces after 28 d fecal microbiota transplantation, and bacterial 16S rRNA gene sequencing analysis revealed that the FMT partly changed the gut microbiota composition of the DLY pigs (Figure 1). The Shannon index showed a significant difference in the FMT group compared to the CON group, but we did not observe any differences in the Simpson and Chao1 indices (Figure 1A). The β-diversity analysis also showed that the bacterial composition was moderately discrepant, and the sample distribution after FMT was more dispersive based on the PCA distance (Figure 1B). Veen analysis showed that the CON group and FMT group identified 821 common OTUs; however, the FMT group resulted in 170 unique OTUs (Figure 1C). The Sankey plot displayed the composition of the microbiota at the phylum and genus levels (Figure 1D). At the phylum level, Firmicutes and Bacteroides were identified as dominant, and Actinobacteriota were significantly reduced in the CON group when compared to the FMT group (Figure 1F). At the genus level, *Lactobacillus*, *Clostridium_sensu_stricto_1*, *Streptococcus*, *Blautia*, *Terrisporobacter*, and *Subdollgranulum* were identified as dominant. The linear discriminant analysis effect size (LEfSe) (LDA score > 3) (Figure 1E) was further used to identify the microbial biomarkers, and the results indicated that the CON group had one biomarker and the FMT group had fifteen biomarkers, while *Sharpea*, *Rikenellaceae_Rc9_gut_group*, and *Lachnospiraceae_NK4A136_group* (which were mainly SCFAs producers) were enriched in the FMT group (Figure 1G). The differences in relative abundance of these bacteria were consistent with the results of the Lefse analysis. Furthermore, the relative abundance of *Prevotella* and *Christensenellaceae_R-7_group* was significantly higher in the FMT group (Figure 1H).

### 3.3. Ningxiang Pig-Derived Microbiota Altered the Serum Metabolism of DLY Pigs

**Figure 2:**
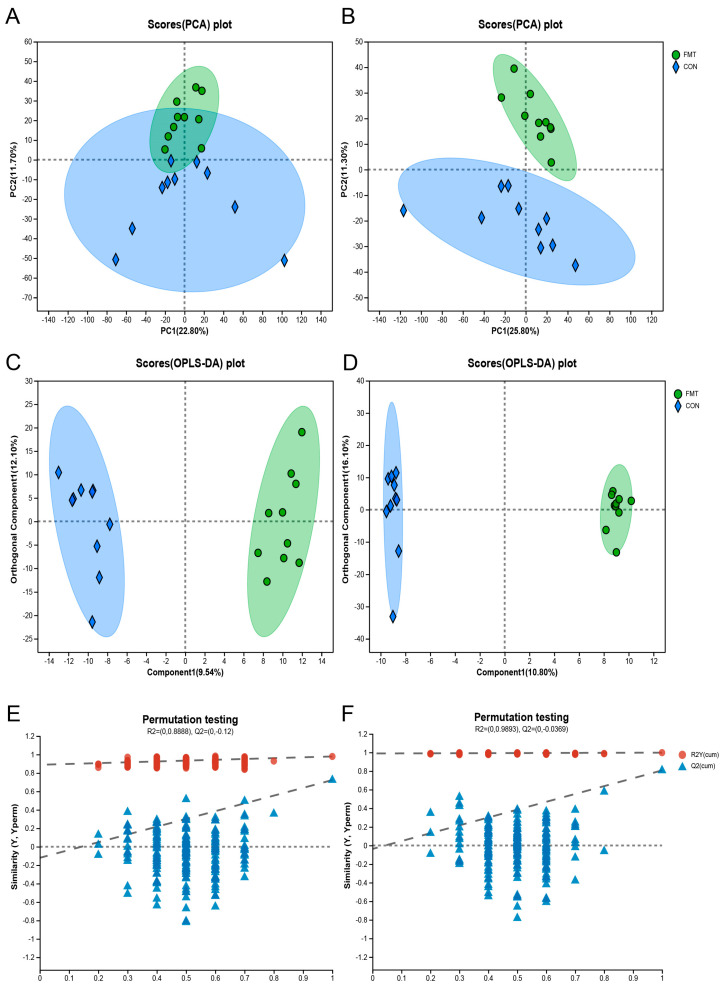
The effect of transplanting Ningxiang pig’s fecal microbiota on the serum metabolic profiles in DLY pigs. (**A**,**B**) Principal Components Analysis (PCA) plots of the metabolites in serum ((**A**) negative ion modes, (**B**) positive ion modes). (**C**,**D**) Orthogonal Partial Least Squares Discriminant Analysis (OPLS−DA) plots of the metabolites in serum ((**C**) negative ion modes, (**D**) positive ion modes). (**E**,**F**) OPLS−DA Permutation Test plots of the metabolites in serum. ((**E**) negative ion modes, (**F**) positive ion modes). The statistical results are shown as means ± SEM (*n* = 10).

**Figure 3:**
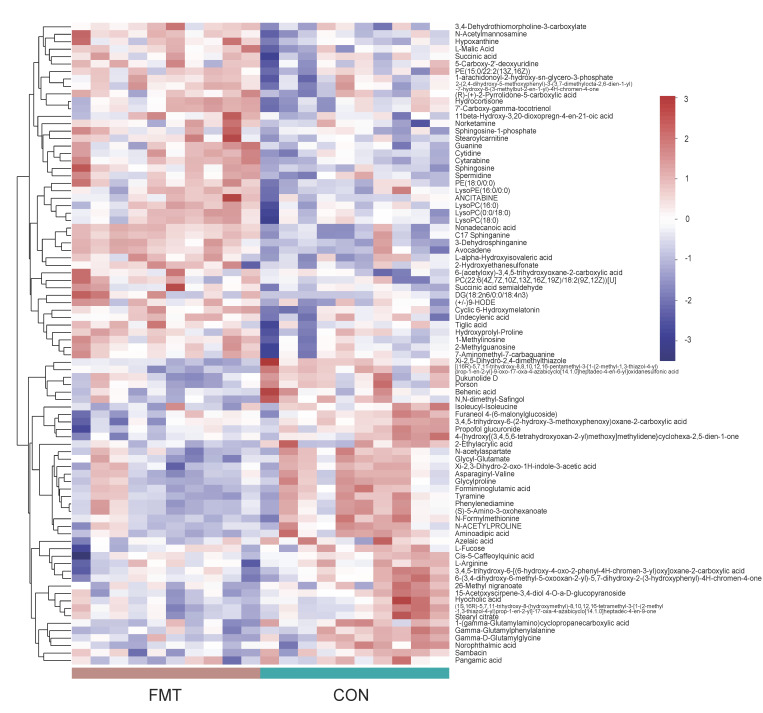
The Hierarchical Clustering Analysis (HCA) of serum differential metabolites that have been identified (Based on the VIP value obtained from OPLS−DA, VIP > 1 and *p* < 0.05 are used as conditions for screening differential metabolites in mix ion modes). The statistical results are shown as means ± SEM (*n* = 10).

**Figure 4:**
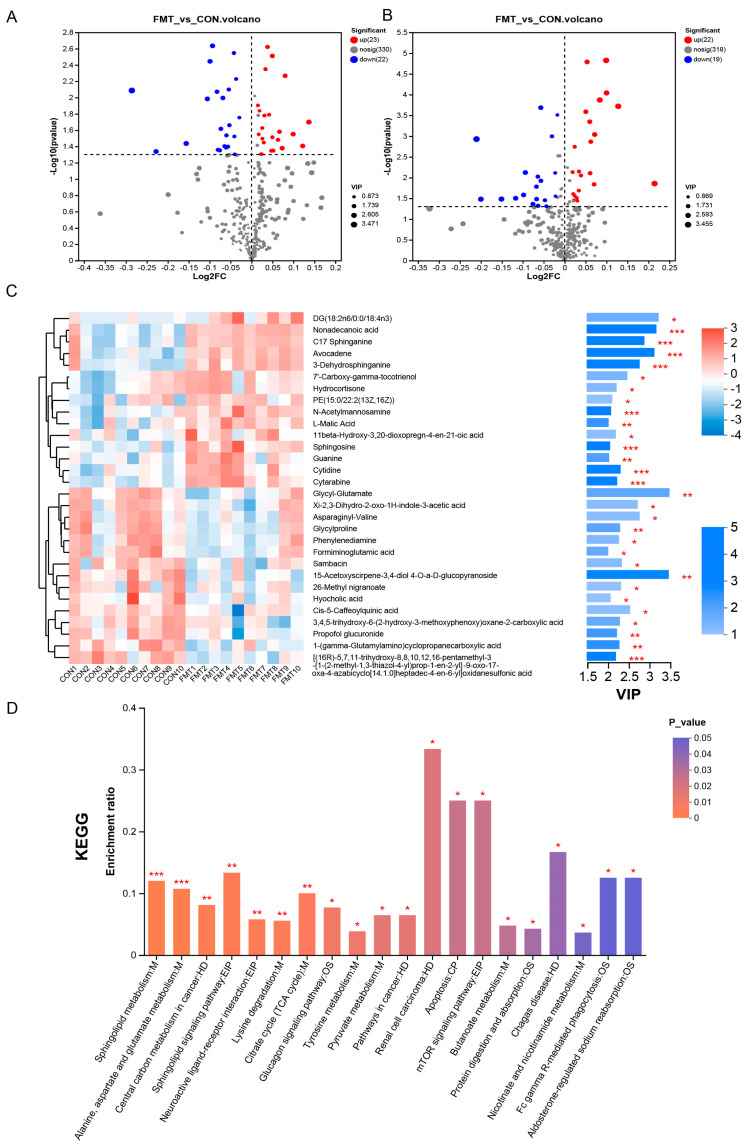
The effect of transplanting Ningxiang pig’s fecal microbiota on the serum metabolic profiles in DLY pigs. (**A**,**B**) The volcano plots of serum metabolites have been identified, with red representing upregulated metabolites and blue representing decreased metabolites ((**A**) negative ion mod, (**B**) positive ion mode) (**C**) Relative abundance and VIP score of the top 30 differentially expressed metabolites (**D**) KEGG pathway enrichment of differentially expressed metabolites. The statistical results are shown as means ± SEM (*n* = 10). (* *p* < 0.05, ** *p* < 0.01, *** *p* < 0.001).

To determine the differential levels of the metabolites in the serum of the fecal microbiota transplanted in the DLY pigs, we performed LC–MS untargeted metabolomics. The Principal Components Analysis (PCA) (Figure 2A,B) and orthogonal partial least squares-discriminant analysis (OPLS-DA) (Figure 2C,D) identified a significant metabolome separation between the serum of the FMT group and the CON group. Meanwhile, the permutation test in both positive ionization mode (R2 = 0.09893, Q2 = −0.0369) and negative ionization mode (R2 = 0.08888, Q2 = −0.12) indicates that the original model has good robustness and there is no overfitting phenomenon (Figure 2E,F). A total of 734 characteristic metabolites were obtained from serum samples collected in the positive and negative ionization modes. According to the *p*-value ≤ 0.05 and VIP > 1 conditions, 86 differential metabolites (positive ionization mode: 41, negative ionization mode: 45) were screened out, and the hierarchical clustering analysis (HCA) of the serum differential metabolites that have been identified (Figure 3) as well as the results of the differential metabolites are shown in the form of volcanic map, in which 45 metabolites (positive ionization mode: 22, negative ionization mode: 23) were significantly increased and 41 metabolites (positive ionization mode: 19, negative ionization mode: 22) were significantly decreased (Figure 4A,B).

Metabolomics results show that there were 86 differential metabolites, mainly belonging to steroids, peptides, organic acids, nucleic acids, lipids, carbohydrates, hormones, and delivery media, that differed between the CON and FMT groups. The top 30 metabolites responsible for the separation between two groups were presented using their relative abundance and VIP scores (Figure 4C). The relative concentrations of DG(18:2n6/0:0/18:4n3), Nonadecanoic acid, C17 Sphinganine, Avocadene, 3-Dehydrosphinganine, 7′-Carboxy-gamma-tocotrienol, Hydrocortisone, PE(15:0/22:2(13Z,16Z)), N-Acetylmannosamine, L-Malic Acid, 11beta-Hydroxy-3,20-dioxopregn-4-en-21-oic acid, Sphingosine, Guanine, Cytidine, and Cytarabine were elevated in the FMT group, while the relative concentrations of Glycyl-Glutamate, Xi-2,3-Dihydro-2-oxo-1H-indole-3-acetic acid, Asparaginyl-Valine, Glycylproline, Phenylenediamine, Formiminoglutamic acid, Sambacin, 15-Acetoxyscirpene-3,4-diol,4-O-a-D-glucopyranoside, 26-Methyl nigranoate, Hyocholic acid, Cis-5-Caffeoylquinic acid, 3,4,5-trihydroxy-6-(2-hydroxy-3-methoxyphenoxy)oxane-2-carboxylic acid, Propofol glucuronide, 1-(gamma-Glutamylamino)cyclopropanecarboxylic acid, and [(16R)-5,7,11-trihydroxy-8,8,10,12,16-pentamethyl-3-[1-(2-methyl-1,3-thiazol-4-yl)prop-1-en-2-yl]-9-oxo-17-oxa-4-azabicyclo [14.1.0]heptadec-4-en-6-yl]oxidanesulfonic acid were decreased in the FMT group.

The pathway enrichment analysis based on the KEGG database showed that metabolites were mainly involved in metabolism, human diseases, environmental information processing, organismal systems, and cellular processes (Figure 4D). Furthermore, the functional pathway analysis of differential metabolites revealed three major enhanced metabolic pathways related to amino acid metabolism, lipid metabolism, and carbohydrate metabolism. Interestingly, Spermidine, Succinic acid semialdehyde, Succinic acid, Sphingosine-1-phosphate, Sphingosine, 3-Dehydrosphinganine, LysoPC(16:0), Hydrocortisone, N-Acetylmannosamine, and L-Malic Acid were enriched in the FMT group, and (S)-5-Amino-3-oxohexanoate, Tyramine, Aminoadipic acid, N-acetylaspartate, L-Arginine, Formiminoglutamic acid, N-Formylmethionine, Behenic acid, and L-Fucose were significantly decreased in the fecal microbiota-transplanted pigs.

### 3.4. Correlation between Serum Metabolites and Fecal Microbiota, Fecal Microbiota, and Digestibility

**Figure 5:**
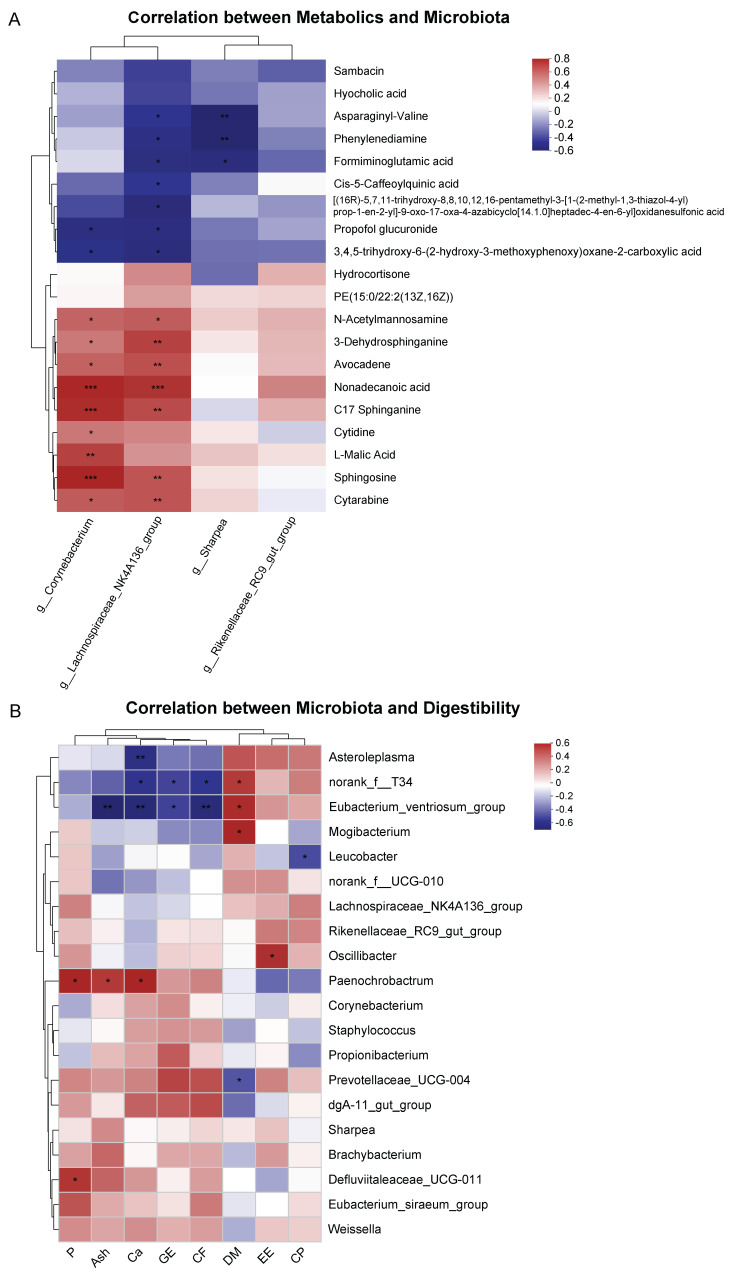
(**A**) Correlation heatmap between differential serum metabolites and differential fecal microbiota. (**B**) Correlation heatmap between fecal microbiota and digestibility. Each grid in the figure represents the correlation between metabolites and microbiota, with red indicating a positive correlation and blue indicating a negative correlation. The depth of the color represents the magnitude of the correlation coefficient. The statistical results are shown as means ± SEM (*n* = 10). (* *p* < 0.05, ** *p* < 0.01, *** *p* < 0.001).

Spearman’s correlation was used to analyze the relationship between the key microbiota (at the genus level, LDA > 3) and the abundance of serum metabolites in the top 20, as well as the relationship between digestibility and the abundance of fecal microbiota in the top 20 (at the genus level) (Figure 5). The positive correlation was between the microbiota *Lachnospiraceae_NK4A136_group*, *Corynebacterium*, and the metabolites N-Acetylmannosamine, 3-Dehydrosphinganine, Avocadene, Nonadecanoic acid, C17 Sphinganine, Cytarabine, and Sphingosine, while the relative abundance of *Corynebacterium* was positively correlated with L-Malic acid and Cytidine (Figure 5A). The relative abundances of *Lachnospiraceae_NK4A136_group* and *Corynebacterium* were negatively correlated with propofol glucuronide and 3,4,5-trihydroxy-6-(2-hydroxy-3-methoxyphenoxy)oxane-2-carboxylic acid, and the relative abundances of *Lachnospiraceae_NK4A136_group* and *Sharpea* were negatively correlated with Asparaginyl-Valine, Phenylenediamine, and Formiminoglutamic acid. Meanwhile, the relative abundance of *Lachnospiraceae_NK4A136_group* was negatively correlated with [(16R)-5,7,11-trihydroxy-8,8,10,12,16-pentamethyl-3-[1-(2-methyl-1,3-thiazol-4-yl)prop-1-en-2-yl]-9-oxo-17-oxa-4-azabicyclo[14.1.0]heptadec-4-en-6-yl]oxidane sulfonic acid and Cis-5-Caffeoylquinic acid. Moreover, Spearman’s analysis was used to examine the correlation between the top 20 fecal microbiota and apparent digestibility. The results reveal that the apparent digestibility of P was positively correlated with the abundance of *Paenochrobactrum* and *Defluviitalaceae_UCG-011*. The apparent digestibility of Ash was positively correlated with *Paenochrobactrum* and negatively correlated with the abundance of *Eubacterium_ventriosum_group*. The apparent digestibility of Ca was positively correlated with *Paenochrobactrum*, and negatively correlated with *Astroleplasma*, *Norank_f_T34*, and *Eubacterium_ventriosum_group*. The apparent digestibility of GE and CF was negatively correlated with *Norank_f_T34* and *Eubacterium_ventriosum_group*. The apparent digestibility of DM was positively correlated with *Norank_f_T34*, *Eubacterium_ventriosum_group*, and *Mogibacterium*. The apparent digestibility of EE was positively correlated with that of *Oscillibacter*. The apparent digestibility of CP was negatively correlated with *Leucobacter* (Figure 5B).

## 4. Discussion

Previous studies have shown that FMT has been recognized as an effective treatment for various diseases, such as IBD and metabolic syndrome, due to its ability to regulate intestinal microorganisms and transfer host phenotypes [14,15]. Ningxiang pigs are characterized by high tolerance to roughage, high fat content, and high stress resistance, which may be related to their microbiota [12,16]. This study aims to investigate the effects of fecal microbiota transplantation from Ningxiang pigs into DLY pigs.

Early intervention based on microbial transplantation can improve the intestinal health of animals thus promoting the growth of animals [17,18]. Similarly, our results showed that FMT significantly promoted the growth performance of DLY pigs. The digestion and utilization of nutrients can reflect the process of animal growth and development. In this study, FMT significantly improved the apparent digestibility of GE, Ash, CF, and Ca in the feces of DLY pigs. The improvement in nutrient apparent digestibility can promote feed conversion efficiency and improve animal growth performance. Studies have shown that the gut microbiota plays an important role in regulating animal nutrient digestibility [19]. Tilg and Kaser [20] showed that the gut microbiota increased obesity mainly by increasing energy ingestion from food and regulating fat deposition. Peter et al. [21] showed that the gut microbiota communities are different between obese and lean individuals. Our results showed that the relative abundance of *Oscillibacter* was positively correlated with apparent EE digestibility, while *Oscillibacter* was significantly correlated with obesity [22]. In addition, our results show that *Norank_f_T34* and *Eubacterium_ventriosum_group* are significantly negatively correlated with CF and GE, and *Eubacterium_ventriosum* is negatively correlated with IBD risk [23]. These results show that FMT has changed the gut microbiota structure of DLY pigs and improved the apparent digestibility of CF and GE. Studies have shown that lactic acid bacteria have the ability to degrade phytase [24]. Phytase can degrade phytate and release chelated Ca^2+^, improving the digestibility of Ca [25]. Our results show that the relative abundance of *Paenochrobactum* was positively correlated with the apparent digestibility of P, Ca, and Ash, suggesting that *Paenochrobactum* may have the ability to degrade phytic acid, thereby promoting the absorption of Ca and P in animals.

Oxidative stress can cause intestinal inflammation, dysbiosis, and diarrhea, and ultimately lead to reduced feed intake and retarded weight gain, which severely affects the economic benefits of farms. Previous studies have revealed that the serum content of MDA, a biomarker of lipid oxidation [26], was significantly increased in weaned pigs [27], while the activity of T-SOD, an antioxidant enzyme, was significantly inhibited [28]. Additionally, CAT is another antioxidant enzyme that aids in eliminating reactive oxygen species (ROS) [29]. Li et al. [30] demonstrated that FMT can be effectively used as a treatment method to eliminate oxidative stress. In this study, FMT reduced the serum MDA level and increased the activities of CAT and T-SOD in the recipient pigs, which was similar to previous studies [18,31]. These results indicate that FMT potentially alleviated oxidative stress in DLY pigs.

The healthy gut microbiota community of pigs possesses diversity, stability, and elasticity; these microorganisms can significantly influence pigs’ physiological, biochemical, and metabolic processes through microbial–host interactions. To investigate whether FMT can regulate the gut microbiota and consequently affect the growth and metabolism of pigs, we sequenced the 16S rRNA gene in the fecal samples. Our findings indicated that FMT intervention changed the diversity of the microbial community and reshaped the composition of gut microbiota in DLY pigs, resulting in significant changes in the abundance of specific bacterial species. Notably, Firmicutes and Bacteroidetes dominate the pig gut microbiota, consistent with previous studies [32,33]. Interestingly, FMT reduced the abundance of Firmicutes in feces [34]. This may be related to diet changes or microbial adaptation changes. Fiber type, for instance, has been shown to influence the abundance of Firmicutes [35]. The relative abundances of *Corynebacterium*, *Sharpea*, *lachnospiraceae_NK4A136_group*, and *rikenellaceae_RC9_gut_group* were enriched in the feces of FMT group. *Sharpea* is associated with complex plant fiber fermentation in mammals (fiber degradation) [36] and is involved in the production and utilization of lactic acid [37], while *Lachnospiraceae_NK4A136_group* and *Rikenellaceae_RC9_gut_group* are related to the production of short chain fatty acids (SCFAs) [38,39,40]. SCFAs are one of the most effective indicators for evaluating intestinal health and can be used as a biomarker to determine the stability of the gut microbiota community [41]. Furthermore, *Prevotella*, known as a dietary fiber fermenter and a potential biomarker of homeostasis [42,43], has garnered significant attention in recent studies. It has been demonstrated that *Prevotella* can augment fat deposition in pigs [44], maintain intestinal health [45], and improve animal growth [46]. Notably, these bacteria were significantly increased in the FMT group, indicating that the relevant beneficial or functional microorganisms have great potential for promoting pig growth, feed conversion efficiency, promoting fat deposition, and improving pig intestinal health. Therefore, it is necessary to improve the composition of the gut microbiota community to improve disease resistance and promote the growth performance of pigs.

Changes in the gut microbiota inevitably trigger corresponding changes in the host metabolic spectrum [11]. As expected, the metabolic characteristics of DLY pigs were changed after the intervention of fecal microorganisms, mainly involving amino acid metabolism, lipid metabolism, and carbohydrate metabolism. FMT significantly increased the lipid metabolism (especially sphingolipid metabolism) of DLY pigs, thereby affecting the changes in the lipid content of DLY pigs. Sphingosine-1-phosphate, a lipid signaling molecule, is mainly produced when the body suffers from inflammation and tissue damage. It plays an important role in cell signal transduction and the mediation of inflammatory and immune responses [47]. Sphingosine can protect the intestinal tract from infection by regulating immune cells [48]. Spearman’s correlation analysis showed that the relative abundance of *Lachnospiraceae_NK4A136_group* and *Corynebacterium* was highly correlated with the changes in lipid compounds. Our results show that FMT alters lipid metabolism in DLY pigs, which also provides a potential explanation for how gut microbiota affects host growth [43].

In addition, FMT also significantly changed the amino acid metabolism and carbohydrate metabolism of pigs, especially tyramine, N-acetylmannosamine, and L-malic acid. Tyramine is a kind of biogenic amine. It shows protective effects on the intestine in a concentration-dependent manner [49]. The reduction in tyramine concentration in DLY pigs caused by FMT may be related to the reduction in Enterobacteriaceae abundance, and members of this family can produce biogenic amines [50]. Furthermore, L-malic acid is an organic acid that plays an important role in improving the antioxidant capacity and oxidative fibers percentage of weaned pigs [51]. It also modulates the gut microbiota of pregnant sows so as to improve the reproductive performance and growth performance of pigs [52]. Our findings further elucidate the beneficial effects of FMT on growth performance and antioxidant capacity of DLY pigs. In summary, this study not only sheds light on the profound impact of gut microbiota on host metabolism but also paves new paths for improving animal health and growth through gut microbiota modulation.

## 5. Conclusions

In general, Ningxiang pig-derived microbiota can improve the growth performance, feed efficiency, and enhance antioxidant capacity of DLY pigs by improving gut microbiota structure and metabolic features. In addition, our study found that *Lachnospiraceae_NK4A136_group* and *Corynebacterium* can be used as potential key microbiota to regulate host lipid metabolism. Therefore, it is necessary to further study the potential mechanism of *Lachnospiraceae_NK4A136_group* and *Corynebacterium* in regulating animal lipid metabolism and clarify the interaction in “microbiota–lipid metabolism”. Although this study has some limitations, these results provide an important reference for improving the antioxidant capacity of animals, regulating the structure of gut microbiota, and promoting the growth of animals.

## Figures and Tables

**Table 1:** Effects of transplanting Ningxiang pig’s fecal microbiota on growth performance of DLY pigs.

Items	CON ^1^	FMT ^2^	*p*-Value
Initial weight (kg)	19.79 ± 0.31	19.58 ± 0.30	0.620
Final weight (kg)	35.41 ± 0.89	38.60 ± 1.31	0.055
ADG (g/d)	558.10 ± 33.87	679.52 ± 47.15	0.046

^1^ CON: control group with basic diet. ^2^ FMT: Fecal microbiota transplantation group with Ningxiang pig’s fecal bacterial suspension added to basic diet. The statistical results are shown as means ± SEM (*n* = 18).

**Table 2:** Effects of transplanting Ningxiang pig’s fecal microbiota on nutrient apparent digestibility of DLY pigs (%).

Items	CON ^1^	FMT ^2^	*p*-Value
DM	86.8 ± 0.09	86.08 ± 0.27	0.039
GE	87.96 ± 0.06	88.65 ± 0.10	<0.001
EE	86.31 ± 0.58	84.6 ± 0.84	0.122
CP	91.33 ± 0.22	90.93 ± 0.15	0.178
CF	42.08 ± 0.83	47.76 ± 1.62	0.011
ASH	64.06 ± 0.53	67.42 ± 0.40	<0.001
Ca	50.59 ± 0.87	55.12 ± 1.56	0.026
P	48.95 ± 0.75	51.77 ± 1.44	0.102

^1^ CON: control group with basic diet. ^2^ FMT: Fecal microbiota transplantation group with Ningxiang pig’s fecal bacterial suspension added to basic diet. The statistical results are shown as means ± SEM (*n* = 10).

**Table 3:** Effects of transplanting Ningxiang pig’s fecal microbiota on the serum antioxidant indexes of DLY pigs.

Items	CON ^1^	FMT ^2^	*p*-Value
CAT (U/mL)	20.00 ± 2.86	30.79 ± 3.66	0.039
TAOC (U/mL)	2.38 ± 0.48	3.58 ± 1.14	0.351
TSOD (U/mL)	56.43 ± 5.05	70.97 ± 3.09	0.034
MDA (nmol/mL)	15.05 ± 3.31	5.21 ± 0.63	0.027

^1^ CON: control group with basic diet. ^2^ FMT: Fecal microbiota transplantation group with Ningxiang pig’s fecal bacterial suspension added to basic diet. The statistical results are shown as means ± SEM (*n* = 10).

## Data Availability

Data are contained within the article and Supplementary Materials.

## Supplementary Materials

The following supporting information can be downloaded at: https://www.mdpi.com/article/10.3390/ani14172450/s1, Table S1: Compositions and nutrient levels of feed (air-dry basis).
