# Supplementary material for: Ningxiang Pig-Derived Microbiota Affects the Growth Performance, Gut Microbiota, and Serum Metabolome of Nursery Pigs
Source: Animals (Basel). 2024 Aug 23;14(17):2450. doi: 10.3390/ani14172450 (PMC11394380; doi:10.3390/ani14172450)
Supplement: Supplementary file 1 [file animals-14-02450-s001.zip › animals-3122485-supplementary.pdf]

Table S1. Compositions and nutrient levels of feed (air-dry basis)

Ingredients	Contents (%)
Corn	57.50
Wheat bran	10.00
Full-fat rice bran	15.00
Soybean meal 43%	14.00
Stone powder	1.20
L-lysine 70%	0.64
DL-methionine	5.02
L-threonine	0.15
Calcium hydrogen phosphate	0.50
Choline chloride 50%	0.10
Salt	0.36
Premix[1)]	0.50
Total	100.00
Nutrient levels[2)]	
Dry matter(%)	86.64
Crude protein(%)	14.64
Crude fat(%)	5.11
Crude fiber(%)	3.94
Ca(%)	0.66
P(%)	0.65
GE(MJ/kg)	16.22
DE(MJ/kg)	13.19
Total lysine(%)	1.00
Total methionine(%)	0.30

[1)]Premix provided the following for per kilogram of the diet: VA 9, 750.00 IU, VD_3 3,000.00 IU, VE 24.00 mg, VK_3 3.00 mg, VB_1 3.00 mg, VB_2 7.50 mg, VB_6 4.50 mg, VB_{12} 30.00 µg, Nicotinamide 36.00 mg, D-Calcium Pantothenate 21.00 mg, Folic acid 1.50 mg, Biotin 0.15 mg, Fe 180.00 mg, Cu 108.75 mg, Mn 59.63 mg, Zn 102.38 mg, I 1.05 mg, Se 0.53 mg.

[2)]Nutrient levels were measured values.
